# Effect of an indwelling nasogastric tube on swallowing function in elderly post-stroke dysphagia patients with long-term nasal feeding

**DOI:** 10.1186/s12883-019-1314-6

**Published:** 2019-05-01

**Authors:** Zhi-Yong Wang, Jian-Min Chen, Guo-Xin Ni

**Affiliations:** 1 Department of Rehabilitation Medicine, First Affiliated Hospital, Fujian Medical University, 20 Chazhong Road, Fuzhou, 350005 China; 2 School of Sports Medicine and Rehabilitation, Beijing Sport University, No. 48 Shangdi Information Road, Beijing, 100084 China

**Keywords:** Indwelling nasogastric tube, Post-stroke dysphagia, Swallowing function, Long-term nasal feeding

## Abstract

**Background:**

In clinical practice, a large number of post-stroke survivors require nasogastric tube (NGT) placement and nasal feeding for a relatively long period. However, its impact on the swallowing function remains largely unknown. This study examines the impact of prolonged placement of an NGT on the swallowing function of elderly post-stroke patients.

**Methods:**

The participants of this study were 30 elderly post-stroke patients who had been using an NGT for more than 2 months. A videofluoroscopic swallowing study (VFSS) was performed before and 5 h after removal of the NGT. The following parameters were analyzed and compared, the functional dysphagia scale (FDS), residue in the valleculae, residue in the pyriform sinuses, and the penetration-aspiration scale (PAS). In addition, prior to the VFSS, the pharynx and larynx were examined using a fiberoptic laryngoscope.

**Results:**

Significant differences were observed between the total scores of the FDS, pharyngeal transit times (PTTs), the residue in the valleculae, and the residue in the pyriform sinuses before and after the NGT removal, suggesting an improved swallowing function following the removal of the NGT. A significantly lower penetration-aspiration degree was found after removing the NGT compared with that before its removal. In addition, examinations using the fiberoptic laryngoscope showed that laryngopharyngeal edema was present in three quarters of the patients.

**Conclusions:**

Our results demonstrate that prolonged placement of the NGT had a negative impact on the swallowing function of elderly post-stroke dysphagia patients, mainly on the pharyngeal phase.

## Background

Stroke is the second leading cause of death and disability worldwide [1]. As one of the potentially fatal complications [2], dysphagia is present in more than 50% of post-stroke survivors [3]. This problem relates to the high risk of post-stroke pneumonia, long length of hospital stay, malnutrition, and even mortality [4]. In clinical practice, a nasogastric tube (NGT)—an easily applied and relatively noninvasive means— is commonly used to provide adequate nutrition and a route for medication administration to patients with dysphagia [5]. However, a significant concern is whether its placement has a negative effect on the swallowing function.

A body of evidence indicates that, for healthy subject, the short-term effects of the NGT on the swallowing function largely depend on the subject’s age and the tube size [6, 7]. Additionally, investigations on acute stroke patient suggest that the NGT does not lead to either increased post-stroke pneumonia incidence and mortality or poor functional outcome [8]. Dziewas et al. compared the swallowing functions of acute stroke patients with and without an NGT in place using fiberoptic endoscopic evaluation of swallowing (FEES), and demonstrated that a correctly placed NGT had little negative effect on the patient’s swallowing function [9]. However, many post-stroke patients require NGT placement and nasal feeding for a relatively long period. Although those patients with prolonged NGT placement are prone to nasal wing lesions, chronic sinusitis, gastro-esophageal reflux, and aspiration pneumonia [10], the impact of prolonged NGT placement on the swallowing function remains largely unknown.

While existing literature has compared the swallowing function before and after the NGT removal to determine the impact of the NGT placement, the examination timing after the NGT removal appears to be an important influencing factor in the effects caused. In a study using videofluoroscopic swallowing study (VFSS) to assess the swallowing function of stroke patients with long-term placement, no significant difference was found in swallowing time before and 30 min after removal of the NGT [11]. Nevertheless, opposite findings were observed in another study comparing the swallowing function of patients with dysphagia immediately and 5 h after the NGT removal. The improved swallowing function 5 h after removal of the NGT [12] indicated that, to accurately assess the swallowing function, NGT-fed patients should rest for a certain period of time following its removal.

Another influencing factor may be the patient’s age. It was reported that the presence of the NGT had little impact on the swallowing function of young healthy adults [7]. However, opposite results were revealed among older healthy individuals [6]. In parallel, a study using a manometric catheter found an age-related effect on the swallowing function [13]. Since elderly patients account for a large proportion of post-stroke patients with dysphagia and long-term nasal feeding [14], it is of great clinical relevance to understand whether and how prolonged NGT placement affects the swallowing function. To address this issue, VFSS was performed before and 5 h after removal of the NGT from elderly post-stroke patients, and several parameters were compared to understand the effect of prolonged NGT placement on their swallowing function.

## Methods

### Participants

Post-stroke patients with NGT were recruited from inpatients in the Department of Rehabilitation Medicine, the First Affiliated Hospital, Fujian Medical University between March 2016 and June 2017. The protocol was approved by the Ethics Committee of the First Affiliated Hospital, Fujian Medical University. The entire study design and procedures were performed in accordance with the Declaration of Helsinki. Written informed consent was obtained prior to participation.

In this study, participants had to fulfill the following inclusion criteria: (1) above 60 years old, (2) stroke-related dysphagia, (3) NGT placement for nutrition supply for at least 2 months, (4) NGT is a fine-bore tube and the same size (CH14–110) as that used by all other participants, and (5) no aspiration during the VFSS. Exclusion criteria were: (1) severely decreased consciousness, (2) a Mini-Mental State Examination (MMSE) score of less than 21, and (3) unstable medical conditions such as severe pneumonia or decompensated congestive heart failure.

### Experimental procedure

Each participant’s swallowing function was assessed before and 5 h after the removal of the NGT using VFSS, using the procedure described previously [15]. The fluoroscopic equipment (Luminosd RF Max, Simens AG, Germany) was used with a pulse rate of 15 pulses per second. During the VFSS, the patient was seated on a chair about 1.2 m away from an X-ray tube, and instructed to maintain an erected lateral position and a neutral head position to obtain a clear viewing of his or her oral cavity, pharynx, larynx, trachea, oesophagus and cervical spine. Thereafter, the patient was asked to swallow the following in a normal manner: (1) 5 ml thin barium sulfate three times, and (2) 5 ml thick barium sulfate three times [11]. In this study, the International Dysphagia Diet Standardisation Initiative (IDDSI) framework was applied to specify the liquid thickness [16]. According to the IDDSI, thin barium sulphate has a viscosity similar to water, equivalent to level 0, whereas thick barium sulphate, similar to nectar, has a viscosity equivalent to level 3. The entire procedure was recorded on video, and analyzed afterwards. The video recordings had a frame rate of 30 frames per second.

On the same day, a fiberoptic laryngoscope was applied prior to the VFSS, using an Olympus laryngoscope (CLV-S40Pro) attached to a color monitor. To observe the physiological change of the pharynx and larynx, a laryngoscope was inserted through the patent’s naris.

### Data processing and analysis

Two investigators (ZY Wang and JM Chen) analysed the VFSS video together. A number of parameters were obtained to provide a quantitative and objective assessment for dysphagia, including the functional dysphagia scale (FDS) [17], the residue in the valleculae and pyriform sinuses, and the penetration-aspiration scale (PAS). For each parameter, scores obtained from three trials were averaged.

To quantitatively measure the oral and pharyngeal functions based on the VFSS video, the FDS was used, which comprised of the 11 following items: lip closure score, bolus formation, residue in the oral cavity, oral transit time (OTT), triggering of the pharyngeal swallow, laryngeal elevation and epiglottic closure, nasal penetration, residue in the valleculae, coating of pharyngeal wall after swallow, and the pharyngeal transit time (PTT). The total score of FDS ranges from 0 to 100, the higher the FDS score, the worse the swallowing function. In addition, the OTT and the PTT were examined according to a method described previously [18].

The PAS is commonly used to assess the degree of penetration-aspiration [12, 19–22]. Penetration is defined as any material entering the laryngeal vestibule not below the vocal folds, whereas aspiration is defined as any material entering the larynx below the vocal folds. In the PAS, the scores range from 1 to 8; the higher the score, the more severe the degree of penetration-aspiration.

In addition, a validated three-point severity scale was applied to assess the residue in the vallecula and pyriform sinuses [23]. The residue was graded on a scale of 0–3, with 0 representing none, 1 representing mild, 2 representing moderate, and 3 representing severe. Residue in either the valleculae or piriform sinuses constituting less than 25% of the height of the structure was defined as mild, between 25 to 50% as moderate, and higher than 50% as severe.

### Statistical analysis

The SPSS software (version 22.0, IMB Corporation, Armonk, NY, USA) was used to analyze all collected data. The quantitative variables were expressed as means ± SD, and a paired t-test was used to confirm the statistical difference of VFSS parameters before and after the NGT removal. A Bonferroni correction was applied wherever necessary. The qualitative variables were expressed as absolute values, and the Wilcoxon signed-rank test was used to compare the statistical difference between the two examinations. The statistical significance was set as *p* < 0.05.

## Results

Of the 37 patients were recruited, 7 were excluded because they did not meet the inclusion criteria, leaving 30 patients that were included in this study. Table 1 presents the participants’ demographics and clinical characteristics. On the day of the experiments, the patients were 71.43 ± 4.06 years old with an average duration of NGT placement lasting 69.47 ± 7.66 days.

**Table 1:** Demographics and clinical characteristics of the subjects

Variables	Value
Age (years old)	71.43 ± 4.06
Sex (male: female)	17:13
Number of patients with ischemic stroke	20
Number of patients with hemorrhagic stroke	10
Time of the NGT placement (day)	69.47 ± 7.66
The total score of the MMSE	24.33 ± 5.07

Table 2 presents the results of the FDS, OTT and PTT with thin and thick barium before and after the NGT removal. Compared with the scores taken before the NGT removal, significantly lower FDS scores were obtained after the NGT removal, indicating an improved swallowing function among patients following the NGT removal. No significant differences were observed in the OTT between before and after the NGT removal; however, the PTT was significantly faster after the NGT removal compared with before the NGT removal. These results suggest that the NGT removal had little effect on the OTT, but led to a significantly faster PTT.

**Table 2:** Results of FDS, OTT and PTT for VFSS before and after the NGT removal

	Before removal	After removal	*P*-value #
FDS score			
Thin Barium	28.07 ± 5.01	12.20 ± 4.39	<0.001
Thick Barium	30.23 ± 6.09	13.93 ± 6.03	<0.001
OTT (second)			
Thin Barium	1.69 ± 0.21	1.67 ± 0.21	1.548
Thick Barium	1.76 ± 0.24	1.73 ± 0.25	0.528
PTT (second)			
Thin Barium	1.48 ± 0.14	1.08 ± 0.16	<0.001
Thick Barium	1.51 ± 0.17	1.09 ± 0.16	<0.001

# Bonferroni corrected P-value

**Fig. 1:**
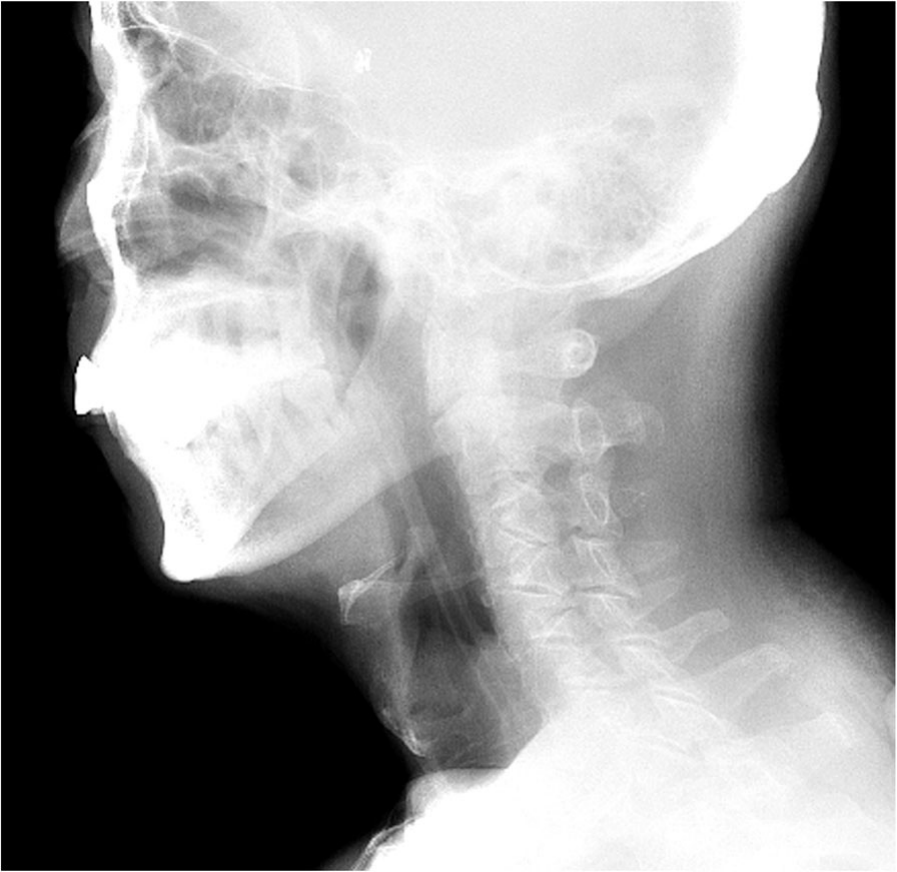
A representative image showing the interference of the nasogastric tube with an epiglottic downward tilt (RED ARROW) during the swallowing process

**Fig. 2:**
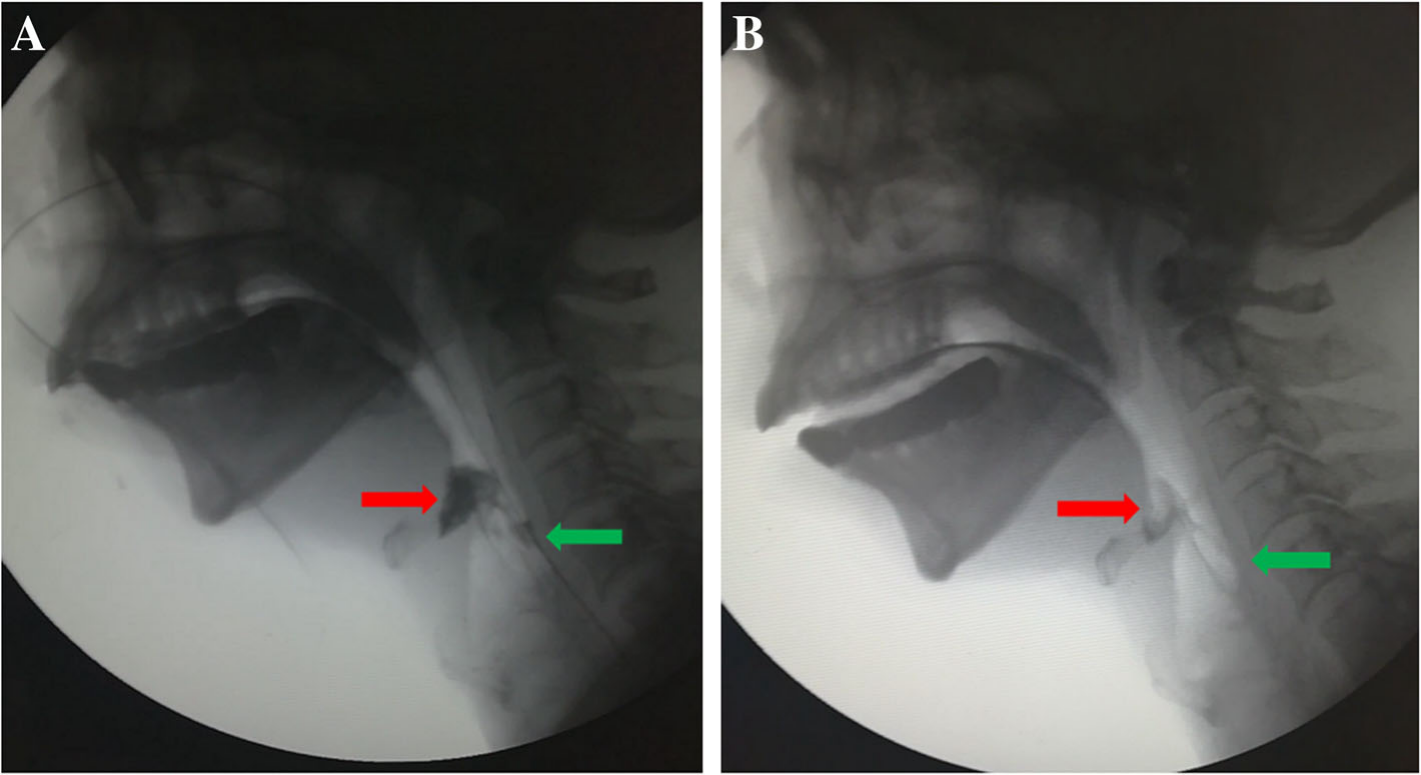
Representative images showing the residues in the valleculae (RED ARROW) and pyriform sinuses (GREEN ARROW) before (**a**) and 5 h after the NGT removal (**b**)

Using VFSS video, abnormal epiglottic movement pattern was observed during the swallowing process, which was associated with the interference of the NGT with epiglottic movement (Fig. 1). In addition, residues were displayed in the valleculae and pyriform sinuses before (Fig. 2a) and after the NGT removal (Fig. 2b), respectively. To further quantity the residues, a validated three-point severity scale was used in this study, and the results are shown in Tables 3. The findings show that, with thin or thick barium, significantly less residues were observed in the valleculae and pyriform sinuses after the NGT removal compared with before the NGT removal. In addition, the PAS was used to assess the degree of penetration-aspiration. Table 4 presents the results of the PAS with either thin or thick barium, showing the number of patients at various scales. A significantly lower degree of penetration-aspiration was found after the NGT removal compared with that before the NGT removal, indicating that NGT placement was associated with the increased penetration-aspiration rating.

**Table 3:** Results of residue in valleculae and pyriform sinuses for VFSS before and after the NGT removal

	Before removal				After removal				Z-value	*P*-value^a^
	0	1	2	3	0	1	2	3		
Valleculae Residue										
Thin Barium	0	0	22	8	8	21	1	0	−3.92	<0.001
Thick Barium	0	0	15	15	4	20	6	0	−2.92	0.003
Pyriform sinuses Residue										
Thin Barium	0	6	23	1	16	14	0	0	−3.391	0.001
Thick Barium	0	2	20	8	4	22	4	0	−2.82	0.005

^a^ Wilcoxon signed-rank test

**Table 4:** Results of PAS for VFSS before and after the NGT removal

	Before removal				After removal				Z-value	*P*-value^a^
	1	2	3	4	1	2	3	4		
PAS										
Thin Barium	19	2	3	6	26	1	3	0	−5.285	<0.001
Thick Barium	19	2	8	1	26	3	1	0	−5.337	<0.001

^a^ Wilcoxon signed-rank test

**Fig. 3:**
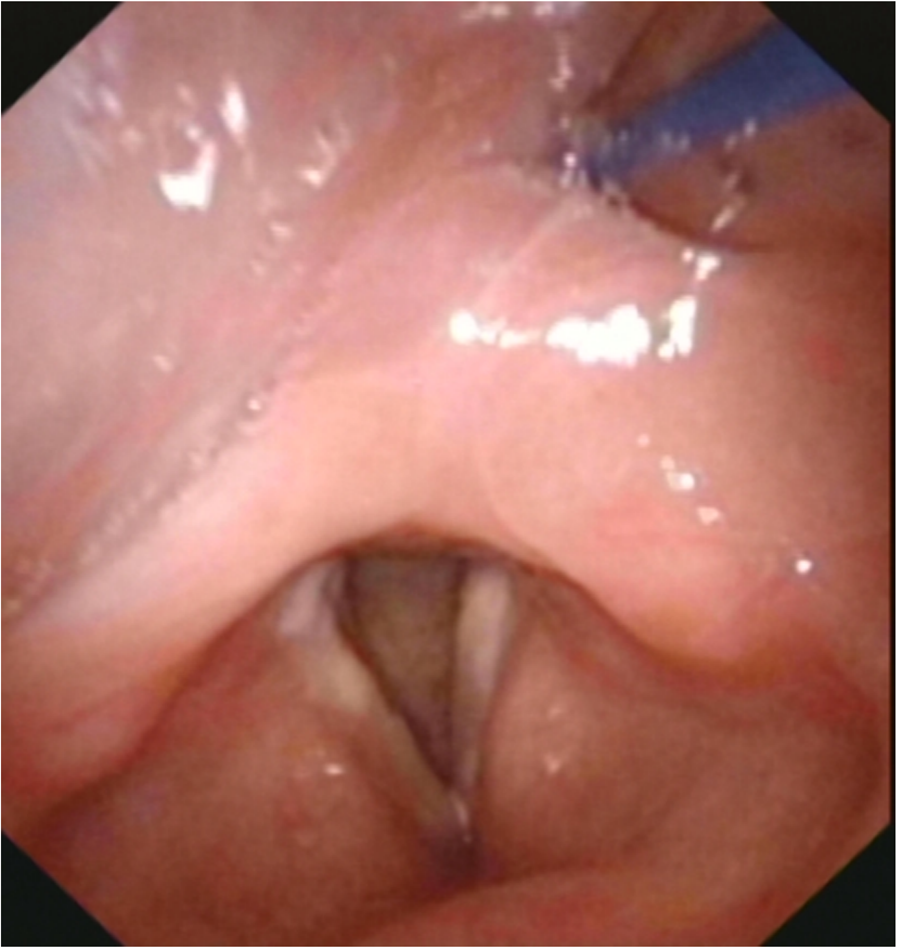
A representative image of the fiberoptic laryngoscope showing laryngopharyngeal edema

Fifteen patients complained of sore throat. Prior to the VFSS, a fiberoptic laryngoscope was applied to all patients. Among them, 25 patients presented various degrees of edema in the pharynx and/or larynx (Fig. 3).

## Discussion

Although NGT is commonly applied to elderly post-stroke patient, the impact of prolonged NGT placement on the swallowing function remains unknown. In this study, VFSS was performed before and 5 h after removal of the NGT, and our findings suggest that prolonged NGT placement has a negative impact on the patient’s swallowing function, mainly on the pharyngeal phase.

The swallowing process typically comprises four stages: the oral preparation phase, the oral phase, the pharyngeal phase, and the esophageal phase [24]. The oral preparation and oral activities are voluntary, while the pharyngeal and esophageal activities are involuntary. Our results suggested that prolonged NGT presence had little effect on the oral preparation and oral phases. During the oral preparation phase, the dysfunction relates mainly to cognitive impairment in stroke patients [25]. Typically, the NGT placement has no connection with the oral preparation phase. Similarly, NGT placement is unlikely to affect the esophageal phase directly, though post-stroke patients with an indwelling NGT may occasionally suffer from gastroesophageal reflux, reflux esophagitis or oesophageal erosion [26]. In parallel, as the NGT is inserted into the stomach through the nostrils, pharyngeal cavity and esophagus, instead of the oral cavity, a non-significant difference was found in the OTT between before and after the NGT removal, indicating that NGT placement has little impact on the oral phase.

Our findings suggest that NGT placement mainly affects the pharyngeal phase. In this work, compared with after the NGT removal, significantly more residues were observed in the valleculae and pyriform sinuses with the NGT in place. During the swallowing process, the epiglottis moves from its resting position and tilts downward rapidly to protect the airway. However, abnormal epiglottic movement pattern caused by the disease, such as a delayed tilt, has been significantly associated with increased residue in valleculae [27]. While this study found a similar phenomenon, the abnormal epiglottic movement pattern was determined to be caused by the NGT, as shown in Fig. 1. Together with our finding of a significant reduction in valleculae residue after the NGT removal, this demonstrated that NGT placement interferes to a certain extent with the movement of the epiglottis cartilage and delayed tilt, thus leading to residue in the valleculae. Following the NGT removal, the PTT and the residues in the pyriform sinuses were reduced during the VFSS, suggesting an improvement in the swallowing function. Notably, rest for a certain period following the NGT removal may play a key role in such improvement. Five hours after the NGT removal, shorter PTTs and less residue in the piriform sinuses were reported compared to immediately after the NGT removal in dysphagia patients [13].

The negative impact of the NGT on the pharyngeal phase is likely due to the following reasons. First, among the 30 patients in this study with long-term NGT placement, 15 complained of throat pain, and 25 presented with varying degrees of edema of the pharynx and/or larynx (Fig. 3). It is supposed that NGT placement could lead to sensory disorders, such as sensory deficits or desensitization in the laryngopharyngeal structures. Laryngopharyngeal sensory deficits are closely related to pharyngeal motor function during swallowing [28], since they may weaken the contraction of the pharyngeal muscle and slow down the pharyngeal muscle response, thus causing piriform sinus and valleculae residues, and prolonging the PTT. Further investigations are warranted to quantify the laryngopharyngeal sensory function using the laryngopharyngeal sensory threshold (LPST) [29]. Further, the NGT—a foreign body indwelling in the pharynx— interferes with the swallowing process by narrowing the esophageal sphincter and making the bolus prone to adhering to the NGT surface, thus further increasing pharyngeal residue. Additionally, the NGT may interfere with the coordinated movements of swallowing-related muscles during the swallowing process. Coordination is necessary for normal swallowing, and delicate discoordination may affect the normal swallowing function [30]. Although delicate discoordination was rarely observed during the VFSS in our study, NGT placement was reported to decrease swallowing coordination [13].

As recommended by several nutritional guidelines, enteral tube feeding is commonly used in post-stroke patients with dysphagia to provide adequate nutrient intake and prevent malnutrition [31–33]. Specifically, NGT feeding is usually chosen for short-term enteral tube feeding [31], whereas, gastrostomy tube feeding is recommended if the swallowing function does not restore within a short period. Although gastrostomy tube feeding is superior to nasal feeding in many aspects [31–33], in clinical practice, NGT feeding is usually maintained for a long period mainly because it is a relatively easy technique to administer. However, long-term nasal feeding causes many complications, such as nasal wing lesions, chronic sinusitis and gastro-esophageal reflux [11]. In this study, a significant difference was found in the degree of aspiration-penetration before and 5 h after the NGT removal, indicating that an indwelling NGT increases the risk of aspiration. Aspiration during the swallowing process can cause aspiration pneumonia, resulting in a declined respiratory function, and an increased mortality rate [26]. As suggested in this work, a prolonged indwelling NGT would lead to increased residue and a longer swallowing time. In this regard, the NGT should be removed as soon as possible once no aspiration occurs during the VFSS. For safety considerations, an oral diet should not be taken until several hours after the removal of the tube. In case the NGT was not expected to be removed in the short term, other feeding routes need to be considered, such as percutaneous endoscopic gastrostomy, and intermittent oro-esophageal tube feeding.

A few limitations should be addressed for improvement and further study. First, the sample size used in this study is relatively small, which may compromise our findings to a certain extend. Second, the examiners were not blinded to the NGT placement given the visibility of the NGT on VFSS images, which may have created bias in the data processing and analysis.

## Conclusions

In this study, VFSS was performed before and 5 h after the removal of an NGT to understand the impact of prolonged placement of an NGT on the swallowing function of elderly post-stroke patients. Our findings showed a clear negative impact of the NGT in the swallowing function, mainly on the pharyngeal phase. The NGT should be removed as soon as possible once no aspiration occurs during the VFSS.

## Abbreviations

FDS: functional dysphagia scale
FEES: fiberoptic endoscopic evaluation of swallowing
IDDSI: International Dysphagia Diet Standardisation Initiative
LPST: laryngopharyngeal sensory threshold.
MMSE: Mini-Mental State Examination
NGT: nasogastric tube
OTT: oral transit time
PAS: penetration-aspiration scale
PTT: pharyngeal transit time
VFSS: Videofluoroscopic swallowing study
